# The Cardiotoxicity Induced by Arsenic Trioxide is Alleviated by Salvianolic Acid A via Maintaining Calcium Homeostasis and Inhibiting Endoplasmic Reticulum Stress

**DOI:** 10.3390/molecules24030543

**Published:** 2019-02-02

**Authors:** Ruiying Wang, Jingyi Zhang, Shan Wang, Min Wang, Tianyuan Ye, Yuyang Du, Xueheng Xie, Jingxue Ye, Guibo Sun, Xiaobo Sun

**Affiliations:** 1 Beijing Key Laboratory of Innovative Drug Discovery of Traditional Chinese Medicine (Natural Medicine) and Translational Medicine, Institute of Medicinal Plant Development, Peking Union Medical College and Chinese Academy of Medical Sciences, Beijing 100193, China; wangruiying2233@yeah.net (R.W.); zhangjingyi707@126.com (J.Z.); wangshan19890625@126.com (S.W.); lily_12506053@163.com (M.W.); yetianyuan2013@163.com (T.Y.); du_yuyang@sina.cn (Y.D.); 2 Key Laboratory of Bioactive Substances and Resource Utilization of Chinese Herbal Medicine, Ministry of Education, Beijing 100193, China; 3 Key Laboratory of Efficacy Evaluation of Chinese Medicine against Glycolipid Metabolic Disorders, State Administration of Traditional Chinese Medicine, Beijing 100193, China; 4 Zhongguancun Open Laboratory of the Research and Development of Natural Medicine and Health Products, Beijing 100193, China; 5 Harbin University of Commerce, Harbin 150028, China; xiexueheng@163.com

**Keywords:** salvianolic acid A, arsenic trioxide, cardiotoxicity, calcium overload, endoplasmic reticulum stress

## Abstract

Arsenic trioxide (ATO) has been verified as a breakthrough with respect to the management of acute promyelocytic leukemia (APL) in recent decades but associated with some serious adverse phenomena, particularly cardiac functional abnormalities. Salvianolic acid A (Sal A) is a major effective component in treating ATO-induced cardiotoxicity. Therefore, the objective of our study was to assess whether Sal A had protective effects by the regulation of calcium homeostasis and endoplasmic reticulum (ER) stress. For the in vivo study, BALB/c mice were treated with ATO and/or Sal A via daily tail vein injections for two weeks. For the in vitro study, we detected the effects of ATO and/or Sal A in real time using adult rat ventricular myocytes (ARVMs) and an IonOptix MyoCam system. Our results showed that Sal A pretreatment alleviated cardiac dysfunction and Ca^2+^ overload induced by ATO in vivo and vitro. Moreover, Sal A increased sarcoplasmic reticulum (SR) Ca^2+^-ATPase (SERCA) activity and expression, alleviated [Ca^2+^]ER depletion, and decreased ER stress-related protein expression. Sal A protects the heart from ATO-induced injury and its administration correlates with the modulation of SERCA, the recovery of Ca^2+^ homeostasis, and the down-regulation of ER stress-mediated apoptosis.

## 1. Introduction

Arsenic-containing compounds have been used as medicines to treat multiple diseases, including cancer, syphilis and malaria, for more than 2000 years [1]. However, these compounds were not used worldwide until the Food and Drug Administration (FDA) approved arsenic trioxide (ATO) for the treatment of relapsed and refractory acute promyelocytic leukemia (APL) [2,3]. The clinical application of ATO is hampered by cardiac toxicity manifesting as QT interval prolongation, torsade de pointes (TdP) or even sudden cardiac death [4,5].

The mechanisms underlying ATO-induced cardiotoxicity are complex and include mainly oxidative stress and calcium overload [6,7]. Several studies have shown that these two mechanisms affect each other, as the excessive production of ROS can inhibit the uptake of Ca^2+^ by the release of Ca^2+^ from the endoplasmic reticulum/sarcoplasmic reticulum (ER/SR), resulting in calcium homeostasis imbalances [8,9]. As previously described in our studies, ATO contributes to Ca^2+^ overload and ROS overproduction, which eventually leads to ER stress and oxidative stress-mediated cardiomyocyte apoptosis [10]. Thus, regulation of calcium homeostasis and inhibition of ROS overproduction may protect from ATO-induced myocardial injury.

**Figure 1:**
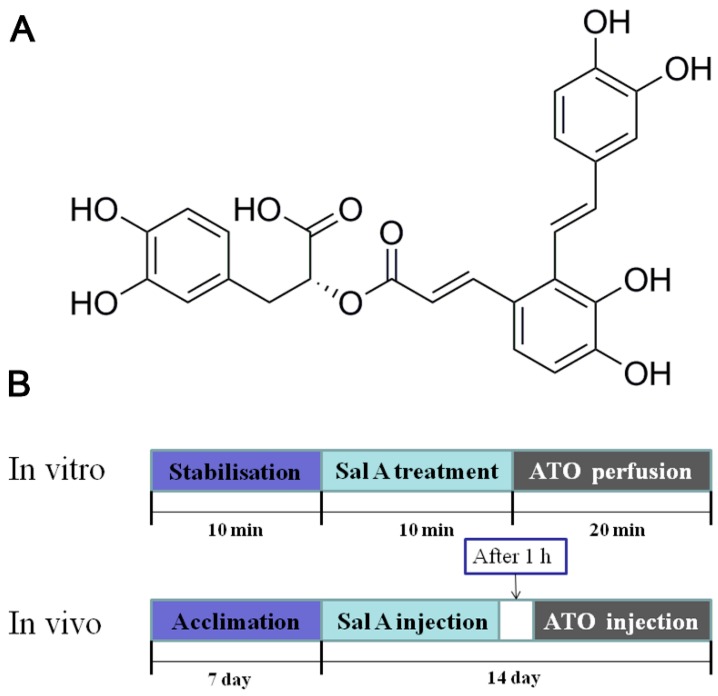
(**A**) Molecular structure of salvianolic acid A (Sal A). (**B**) The in vivo and in vitro experimental designs. ATO, arsenic trioxide.

*Salvia miltiorrhiza* Bunge (also known as Danshen) is widely used in China to treat cardiovascular diseases. Salvianolic acid A (Sal A; Figure 1) is the main effective, water-soluble constituent of *S. miltiorrhiza*, a well-known Chinese traditional herbal medicine. Sal A is able to increase antioxidant enzyme activity, suppress ROS overproduction, and attenuate ATO-induced cardiac injury in H9c2 cells as previously disclosed [11]. It has also been reported that Sal A inhibits I_CaL_ dose-dependently, suggesting that the Ca^2+^-antagonizing effect of Sal A is beneficial with respect to the treatment of myocardial ischemia-reperfusion injury [12]. Moreover, Sal A attenuated ER stress by preventing calcium release in liver LO2 cells [13]. These findings have prompted us to study whether the cardioprotective effects of Sal A are relevant with regulation of calcium homeostasis imbalances and subsequent ER stress-related apoptosis.

Attenuating ATO-induced toxic effects in the heart may have a tremendous impact on anti-cancer treatment. Therefore, this study aimed to demonstrate whether Sal A improves the cardiac contractile functional abnormalities induced by ATO and to investigate the potential effects of Sal A on calcium homeostasis imbalances and ER stress.

## 2. Results

### 2.1. Effects of Sal A on Cardiac Function

**Figure 2:**
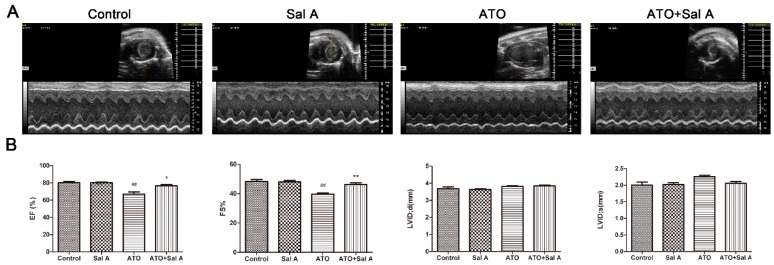
Sal A ameliorated left ventricular functions after ATO treatment. After mice were treated with saline or ATO with/without Sal A pretreatment for two weeks, echocardiography was finished. (**A**) Images of M-mode echocardiograms; (**B**) echocardiography indices, expressed as the mean ± SD. EF, ejection fraction; FS, fractional shortening; LVIDd, left ventricular internal diameter in diastole; LVIDs, left ventricular internal diameter in systole. ^##^ *p* < 0.01 vs. control; * *p* < 0.05 vs. ATO group; ** *p* < 0.01 vs. ATO group.

The experiment evaluated the effect of Sal A through an in vivo mouse model of ATO-induced cardiotoxicity. As grouped, M-mode echocardiography was used to examine the effects (Figure 2A). Both the ejection fraction (EF) and the fractional shortening (FS) were significantly decreased after mice were treated with ATO alone but were dramatically increased after mice were administered the two drugs compared with mice of the control group (Figure 2B). However, systole and diastole functions of the left ventricular showed no significant differences among the four groups.

### 2.2. Sal A Prevents ATO-Induced Myocardial Damage

**Figure 3:**
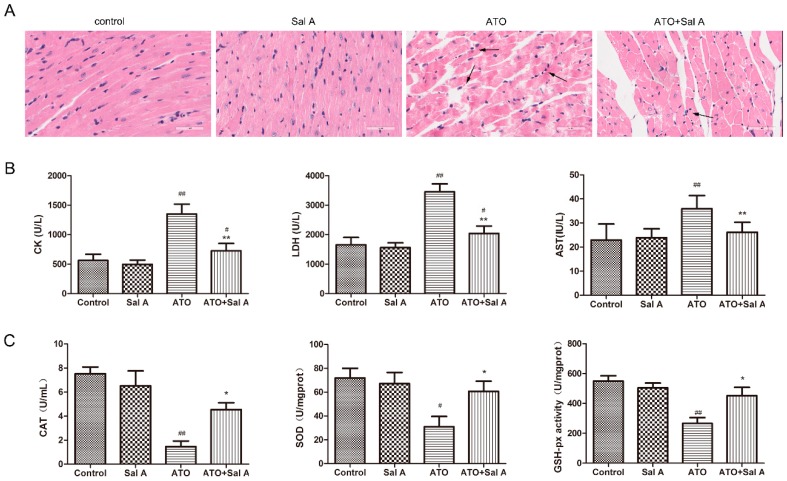
Sal A alleviated ATO-induced myocardial injury in mice hearts. (**A**) Hematoxylin-eosin (HE) staining showed the effects of Sal A on histological changes of mouse hearts. The scale bar is 50 μm. (**B**) Effects of Sal A on creatine kinase (CK), lactate dehydrogenase (LDH), and aspartate aminotransferase (AST) activity in plasma, and (**C**) effects of Sal A on catalase (CAT), superoxide dismutase (SOD), and glutathione peroxidase (GSH-PX) activity in plasma, expressed as the mean ± SD (*n* = 15 per group). ^#^ *p* < 0.05 vs. control; ^##^ *p* < 0.01 vs. control; * *p* < 0.05 vs. ATO group; ** *p* < 0.01 vs. ATO group.

The overall distribution of myocardial damage at the light microscopy level is shown in Figure 3A. The hearts after ATO treatment by hematoxylin-eosin (HE) staining indicated myofibrillar loss, cardiomyocyte necrosis and structural abnormalities, but these abnormalities were partially prevented by Sal A treatment. The Sal A-treated group showed no difference compared to the control group.

The serum levels of cardiac enzymes, including creatine kinase (CK), aspartate aminotransferase (AST), and lactate dehydrogenase (LDH) were measured to reflect myocardial damage [14]. The ATO + Sal A group significantly alleviated the increases of cardiac enzyme levels induced by ATO, while Sal A treatment alone did not induce clear changes in cardiac enzyme levels compared with the control group (Figure 3B). 

### 2.3. Sal A Improves Antioxidant Enzyme Activities

In contrast with the control group, catalase (CAT), glutathione peroxidase (GSH-PX) and superoxide dismutase (SOD) activity levels in the ATO group were decreased. However, this decrease was reversed by the ATO + Sal A group, as shown in Figure 3C. These findings illustrated that Sal A significantly improves antioxidant activity of cardiomyocytes against oxidative stress induced by ATO.

### 2.4. Effects of Sal A on Cardiomyocyte Contractile Function in ARVMs after ATO Treatment

**Figure 4:**
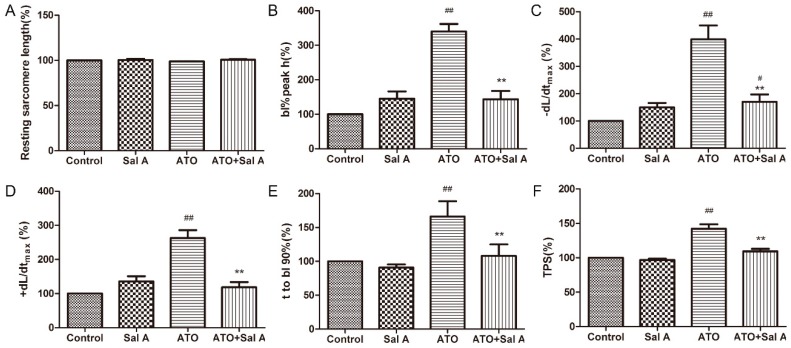
Sal A enhanced contractile function of adult rat ventricular myocytes (ARVMs) after ATO treatment. (**A**) Resting sarcomere length. (**B**) Sarcomere-shortening amplitude. (**C**) maximal relengthening velocity (−dL/dtmax). (**D**) maximal shortening velocity (+dL/dtmax). (**E**) time to 90% relengthening. (**F**) time to peak shortening (TPS). Data are expressed as the mean ± SD (*n* = 30–40 per group), ^#^ *p* < 0.05 vs. control, ^##^ *p* < 0.01 vs. control, ** *p* < 0.01 vs. ATO.

Adult rat ventricular myocytes (ARVMs) were perfused with 1 μM Sal A for 10 min before being treated with 100 μM ATO for 20 min to explore whether the injuries of cardiomyocyte contractile function induced-ATO were alleviated by Sal A. As shown in Figure 4, Sal A treatment did not change six indicators of cardiomyocyte function compared with control treatment. Treatment with ATO + Sal A displayed a normal sarcomere-contraction amplitude (Figure 4B), maximal shortening velocity (+dL/dt) (Figure 4D), time to 90% relengthening (TR90) (Figure 4E), and time to peak shortening (TPS) (Figure 4F), whereas the group treated with ATO displayed a significantly increased sarcomere-shortening amplitude, ±dL/dt, TR90 and TPS compared with the groups treated with other agents. The above results show that ATO treatment severely impaired cardiomyocyte contractile function and that this impairment was eliminated by Sal A treatment.

### 2.5. Effects of Sal A on Intracellular Ca^2+^ Transients in ARVMs after ATO Treatment

**Figure 5:**
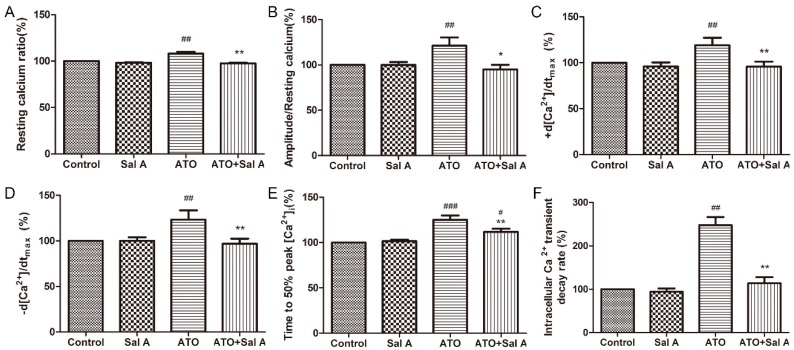
Sal A regulated intracellular Ca^2+^ transients in ARVMs after ATO treatment. (**A**) Resting Ca^2+^ ratio. (**B**) Amplitude/resting calcium ratio. (**C**) Maximal Ca^2+^ shortening velocity (+d[Ca^2+^]/dtmax). (**D**) Maximal Ca^2+^ relaxation velocity (−d[Ca^2+^]/dtmax). (**E**) Time to 50% peak [Ca^2+^]_i_. (**F**) Intracellular Ca^2+^ transient decay rate. Data are expressed as the mean ± SD (*n* = 30–40 per group), ^#^ *p* < 0.05 vs. control, ^##^ *p* < 0.01 vs. control, * *p* < 0.05 vs. ATO, ** *p* < 0.01 vs. ATO.

In our subsequent experiments, Ca^2+^ transients were detected by intracellular fura-2 fluorescence. Similar to the results of the previous study [15], the results of this study showed that ATO treatment induced an increase in the resting Ca^2+^ ratio, Ca^2+^ transient amplitudes, ±d [Ca^2+^]/dtmax, the time to 50% peak [Ca^2+^]_i_, and the [Ca^2+^]_i_ transient decay rate compared with the control group in Figure S1 and Figure S2. Sal A treatment remarkably decreased these indices in the ATO + Sal A group compared with the ATO group (Figure 5A–F). Our data also showed that calcium homeostasis imbalance induced by ATO in ARVMs was greatly ameliorated by Sal A.

### 2.6. Effects of Sal A on sarco endoplasmic reticulum Ca^2+^-ATPase (SERCA) Activity

**Figure 6:**
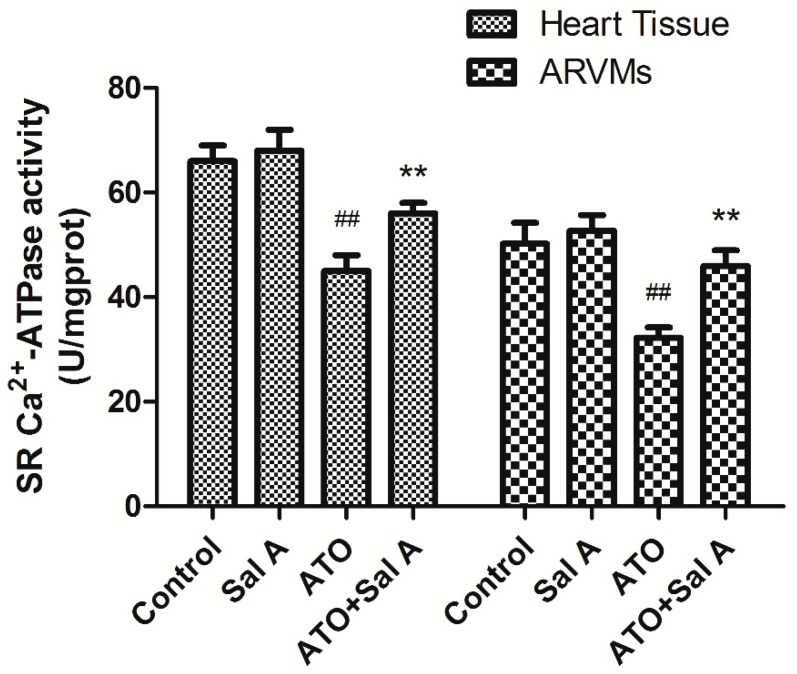
Sal A enhanced SERCA activity in ARVMs and mice heart tissue after ATO treatment. Data are expressed as the mean ± SD; ^##^*p* < 0.01 vs. control, ** *p* < 0.01 vs. ATO.

SERCA2a regulates Ca^2+^ in the intracellular by affecting SR Ca^2+^ intake. Intracellular Ca^2+^ overload and cardiac myocyte dysfunction can be regulated by reducing SERCA expression and activity [16]. SERCA activity of ARVMs and mice heart tissue were detected respectively. ATO treatment reduced SERCA activity compared with the control treatment, whereas Sal A treatment significantly reversed the effects of ATO on SERCA activity both in ARVMs and mice heart tissue in Figure 6. These data suggest that Sal A enhanced SERCA activity so reduced cardiotoxicity induced by ATO.

### 2.7. Effects of Sal A on Ca^2+^ and ER Stress-Related Protein Expression Levels after ATO Treatment

**Figure 7:**
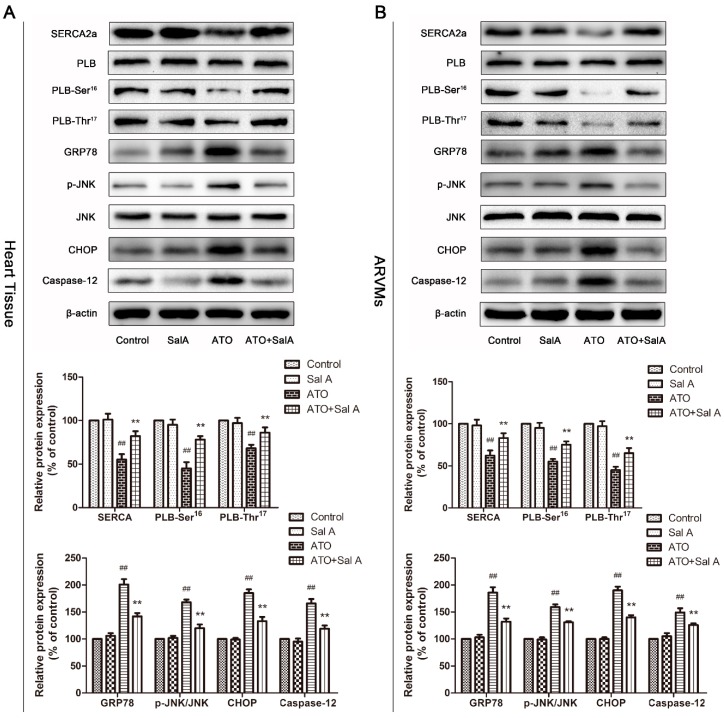
Sal A regulated Ca^2+^-handling and endoplasmic reticulum (ER) stress-related protein expression after ATO treatment. (**A**) Ca^2+^-handling and ER stress-related protein expression in mice heart tissue and (**B**) in ARVMs. All data are expressed as the mean ± SD; ^##^ *p* < 0.01 vs. control, ** *p* < 0.01 vs. ATO.

The results showed the regulatory effects of Sal A-treatment on Ca^2+^-related protein expression in heart tissue and ARVMs. As shown in Figure 7, SERCA2a activity levels and phospholamban (PLB) phosphorylation levels decreased significantly after ATO treatment compared with the control treatment. However, Sal A treatment prevented the decrease. These results indicate that Sal A regulates Ca^2+^-related protein expression levels to prevent calcium overload induced by ATO.

Several studies have confirmed that calcium steady-state imbalances cause calcium overload, which ultimately triggers ER stress [17]. Thus, we examined the expression of ER stress and apoptosis-involved proteins. As shown in Figure 7, expression levels of glucose-regulated protein 78 (GRP78), an ER stress response marker, were markedly higher after ATO treatment than after the control treatment. The same patterns of change were observed for c-Jun NH2-terminal kinase (JNK), C/EBP-homologous protein (CHOP) and caspase-12. However, Sal A significantly attenuated ATO-induced overexpression of ER stress-related proteins in both ARVMs and mice heart tissue. Collectively, the data showed that ER stress was activated after ATO treatment. Most importantly, these results also indicated that Sal A treatment significantly inhibited ER stress and apoptosis induced by ATO.

## 3. Discussion

ATO is a potent treatment for APL; however, due to the cardiotoxicity of ATO, many patients with cancer are unable to receive more efficient treatment [18]. Previous research by our team has shown that salvianolic acid B (Sal B) had a protective effect against cardiotoxicity induced by ATO [19]. Subsequently we found that Sal A also suppresses ATO-induced cardiac dysfunction in vivo and in vitro. Importantly, this study has verified that Sal A effectively attenuated cardiac systolic function and intracellular Ca^2+^ dysfunction induced by ATO. Moreover, SERCA activity in ARVMs and heart tissue increased after the treatment of Sal A compared with the ATO treatment group. The data collected in this study, as well as the data collected in our previous studies, demonstrated that Sal A exerts its cardioprotective effect not only through its antioxidative and anti-apoptotic properties but also through the regulation of calcium homeostasis and ER stress [20]. 

Oxidative stress is widely considered as one of the main mechanisms underlying the toxicity of ATO [19]. We previously showed that Sal A decreased oxidative stress and intracellular ROS levels, and increased antioxidant enzyme activity against ATO in vitro [11]. In this study, consistent with previous studies, Sal A also depressed ATO-induced oxidative stress by increasing the activity of SOD, CAT, GSH-px in vivo. In addition to causing oxidative damage, ATO treatment also caused changes in cardiac function according to the results of echocardiography, and caused myocardial damage by increasing the level of CK, LDH, AST; however, Sal A pretreatment was effective in preventing these alterations and inhibiting cardiotoxicity induced by ATO.

Calcium overload is another primary factor to cause cardiotoxicity by ATO in addition to oxidative stress [11]. Ca^2+^ is a crucial secondary messenger in ventricular myocytes [21]. Ca^2+^ homeostasis and Ca^2+^-mediated signaling are involved in excitation-contraction (EC) coupling and play an important role in normal heart function [22]. ATO treatment leads to a dramatic rise in [Ca^2+^]_i_ due to abnormal Ca^2+^ handling. Here, the results show that ATO-induced cardiac dysfunction is significantly attenuated by Sal A. Moreover, our results showed that Sal A pretreatment relieved ATO-induced decreases in the decay rates of [Ca^2+^]_i_ transient, which demonstrated that Sal A increased SERCA activity. SERCA is the critical regulator of Ca^2+^ homeostasis and contractile activity in the cardiac SR [23]. Our results showed that Sal A preserved SERCA activity in vivo and in vitro and increased SR Ca^2+^ content, which explains how Sal A-maintained [Ca^2+^]_i_ homeostasis. In addition, PLB, as an SR membrane protein, can regulate SERCA function, and Ser16 and Thr17 are its phosphorylation sites [24]. SERCA activity is restrained by dephosphorylated PLB, but is recovered by phosphorylated PLB. Sal A upregulated ATO-mediated decreases in the expression of SERCA and p-PLB in ARVMs and heart tissue. Consequently, ATO-induced Ca^2+^ homeostasis imbalance was alleviated after Sal A treatment by adjusting SERCA activity and expression.

The ER or SR in cardiomyocytes play a critical role in protein synthesis, protein folding and transporting, cellular responses to stress, and the change of Ca^2+^ levels [24]. Firstly, ER transmembrane sensors monitor the unfolded proteins’ accumulation, and activate relative pathways to deal with the unfolded and/or misfolded proteins [25]. For example, GRP78 oligomerizes unfolded proteins and disposes them [26]. However, prolonged or severe ER stress will lead to the apoptosis signaling pathway. It is reported that CHOP is a crucial pro-apoptotic transcription factor in ER-mediated apoptosis pathways. In addition, phosphorylated JNK and activated Caspase-12 are also vital parts in this process [27,28]. ATO treatment induces Ca^2+^ overload and calcium homeostasis imbalances, resulting in ER stress [28]. In this study, we found that ATO upregulated the expression of GRP78, JNK, CHOP and caspase-12, which were ER stress- and apoptosis-related proteins, and that Sal A treatment significantly attenuated these increases. These findings proved that ATO induces ER stress and, then, myocardial cell apoptosis. In addition, Sal A reversed ATO-induced structural and functional abnormalities in ARVMs and mice heart tissue, suggesting that the cardioprotective effects of Sal A against ATO may be related to the inhibition of ER stress.

In summary, Sal A suppresses ATO-induced cardiac dysfunction in vivo and in vitro by decreasing cardiac enzymes levels, increasing antioxidant enzyme activities, maintaining calcium homeostasis and inhibiting endoplasmic reticulum stress. Therefore, all findings serve as evidence showing that Sal A has cardioprotective effects against ATO-induced cardiotoxicity.

## 4. Materials and Methods 

### 4.1. Materials and Animal Treatment

Sal A (content ≥ 98%) was obtained from Shanghai Winherb Medical S&T Development Co., Ltd. (Shanghai, China). ATO was obtained from Harbin YI-DA Pharmaceutical Ltd. (Harbin, China).

Male BALB/c mice (18 to 20 g) and male Sprague–Dawley rats (180–200 g) were bought from Vital River Laboratories, Beijing, China. The protocol was approved by the local animal committee. All procedures and interventions were approved by the Institutional Animal Use and Care Committee (registration number: #IMPLAD2016030715). Sixty mice were randomly divided into 4 groups as following: (a) a control group (administered 10 mL/kg/day saline by intraperitoneal injection 1 h before 10 mL/kg/day saline by tail vein injection), (b) a Sal A group (administered 3 mg/kg/day Sal A by intraperitoneal injection 1 h before 10 mL/kg/day saline by tail vein injection), (c) an ATO group (administered 10 mL/kg/day saline by intraperitoneal injection 1 h before 2 mg/kg/day ATO by tail vein injection), and (d) an ATO+Sal A group (administered 3 mg/kg/day Sal A by intraperitoneal injection 1 h before 2 mg/kg/day ATO by tail vein injection). Two weeks of administration was implemented in the study.

### 4.2. Echocardiographic Measurements 

Echocardiography was used to analyze Cardiac function about 24 h after last drug treatment. The mice were anesthetized using 1.5–2% isoflurane, and the Vevo 770 micro-ultrasound system (VisualSonics, Toronto, ON, Canada) was used to detect and analyze M-mode ultrasound images, and the change of ejection fraction (EF), fractional shortening (FS), left ventricular internal diameter in diastole (LVIDd) and left ventricular internal diameter in systole (LVIDs).

### 4.3. Heart Histopathological Measurements 

After the detection of echocardiography, the mice heart was trimmed and embedded in paraffin blocks after fixation with 4% paraformaldehyde. The hearts were sliced and then stained with hematoxylin and eosin (HE). The images were obtained by a light microscope (CKX41, 170 Olympus, Tokyo, Japan).

### 4.4. Serum Analysis of LDH, CK, AST, GSH-PX, CAT, and SOD

After the detection of echocardiography, blood samples were collected from the fundus vein using a capillary tube while the animals were under anesthesia. After at least 2 h standing, the serums were obtained by centrifuging at 3000 rpm for 15 min. The activity levels of LDH, CK, AST, GSH-PX, CAT, and SOD in serum were detected using commercially available kits purchased from the Jiancheng Bioengineering Institute (Nanjing, China), according to the manufacturer’s instructions.

### 4.5. Isolation of Adult Rat Ventricular Myocytes (ARVMs) 

ARVMs were isolated using the enzymolysis method, as previously reported [10]. In brief, Sprague-Dawley (SD) rats were anesthetized under pentobarbital sodium (140 mg/kg ip) before opening the chest, and the hearts were excised quickly and then suspended on a Langendorff perfusion apparatus. Then the hearts were successively perfused with solution A (HEPES, 10; NaCl, 137; glucose, 10; KCl, 5.4; MgCl_2_, 1.2; and CaCl_2_, 1.2 [in mM]) for 2 min, solution B (HEPES, 10; NaCl, 137; glucose, 10; KCl, 5.4 and MgCl_2_, 1.2 [in mM]) for 5 min, and solution B with collagenase type II for 10–15 min. After perfusion, the single cell was obtained by filtering digested heart tissues using a 300-mm nylon mesh. The cell suspension was deposited 3 times with solution A to progressively increase the extracellular Ca^2+^ concentration to 1.2 mM. Suspensions with a cell viability rate over 85% were used in this study, of which rod-shaped ARVMs with prominent sarcomere were contained.

### 4.6. Measurement of Sarcomere Shortening and Ca^2+^ Transients in ARVMs

Our experiment used video-based sarcomere contractility and calcium recording module in a SoftEdge MyoCam System (IonOptix Corporation, Milton, MA, USA) to assess sarcomere shortening and Ca^2+^ transients, as previously reported [11]. Firstly, isolated ARVMs were incubated with Fura-2/AM (2 μM) for 15 min, then the cells were washed twice with solution A. Subsequently, the ARVMs were placed in a Warner chamber mounted on the stage of an inverted microscope (Olympus, IX-70) and perfused with different solutions at a rate of 1.5 mL/min. The experiment was grouped as follows: (a) control group: solution A for 30 min; (b) Sal A group: solution A with 1 μM Sal A for 10 min; (c) ATO group: solution A with 100 μM ATO for 20 min; and (d) ATO+Sal A group: solution A with 100 μM ATO for 20 min after solution A with 1 μM Sal A for 10 min. The ARVMs were allowed to stabilize for approximately 10 min before being analyzed. In our studies, 30–40 cells from 5 rats per group were obtained. The recording and analysis of data were conducted using IonWizard software (version 6.2.0.59, Milton, MA, USA).

### 4.7. SERCA Activity Detection 

SERCA activity of ARVMs and mice heart tissue were detected respectively. For ARVMs, the rod-shaped cardiomyocytes with prominent sarcomere were treated as followed: (a) control group: solution A for 30 min; (b) Sal A group: solution A with 1 μM Sal A for 10 min; (c) ATO group: solution A with 100 μM ATO for 20 min; and (d) ATO+Sal A group: solution A with 100 μM ATO for 20 min after solution A with 1 μM Sal A for 10 min. After all treatment, ARVMs were added to PBS buffer solution and then broken using ultrasound. Cell suspension was centrifuged for 10 min at 3500 rpm and the supernatant was used to detect the SERCA activity. For mice heart tissue, the tissue of 4 groups was added to 10 mL/g saline, and then fully homogenized. Tissue suspension was centrifuged for 10 min at 3500 rpm and the supernatant was used to detect the SERCA activity. SERCA activity was measured using a commercially available kit (CAT#A070, Nanjing Jiancheng Bioengineering Institute, Nanjing, China). The specific operation was completed according to the manufacturer’s instructions.

### 4.8. Western Blot Analysis

After exposure to the group-specific solutions described in 4.1 and 4.6, the proteins of the mice heart tissue homogenates and ARVMs were obtained with the tissue or cell protein extraction reagent, and were determined by a BCA kit (Pierce Corporation, Rockford, IL, USA). Approximately 30 to 50 μg of protein was loaded onto 10% or 12% sodium dodecyl sulfate (SDS) polyacrylamide gel and then transferred to nitrocellulose membranes, which were incubated overnight at 4 °C with primary antibodies including: Santa Cruz: PLB (sc-21923), PLB-Ser16 (sc-12963), PLB-Thr17 (sc-17024), p-JNK (Thr183/Tyr185, sc-293136), and JNK (sc-7345); Abcam: SERCA (ab2861), caspase-12 (ab18766), GRP78 (ab21685) and CHOP (ab11419). Then the membranes were washed and incubated with secondary antibodies (CWbiotech Beijing, China) for 2h at room temperature. The immunoblots were developed using an electrochemiluminescence (ECL) kit, and then scanned; densitometric analysis was performed using Gel Pro software (Media Cybernetics, Rockville, MD, USA).

### 4.9. Statistical Analysis

All data are presented as means ± standard deviation (SD) from at least 3 independent experiments. Comparisons between different groups were performed using one-way ANOVA followed by Tukey’s test with GraphPad Prism 5.0 software (SPAA Inc., Chicago, IL, USA), and graphical presentation of data was also undertaken with GraphPad Prism. A *p*-value less than 0.05 was considered statistically significant.

## 5. Conclusions

Sal A treatment before ATO suppressed Ca^2+^ overload and improved SERCA activity and expression, thereby alleviating ATO-induced calcium homeostasis imbalances. In addition, Sal A inhibited ATO-induced ER stress by downregulating GRP78, JNK, CHOP and caspase-12. Our findings suggest that Sal A protects against ATO-induced cardiac injury and improves myocardial performance. The cardioprotective effect seems to be due largely to the maintenance of calcium homeostasis and the attenuation of ER stress-associated apoptosis in vivo and in vitro. These results demonstrated that Sal A prevents ATO-induced cardiotoxicity, which has significant implications for clinical application. However, our study is still in its initial stages, and the exact mechanisms underlying the cardioprotective activity of Sal A require further study.

## Supplementary Materials

The following are available online at http://www.mdpi.com/1420-3049/24/3/543/s1, Figure S1: Sal A regulated intracellular Ca^2+^ transients in ARVMs after ATO treatment in dot plots, Figure S2: Representative traces of sarcomere shortening and Ca^2+^ transient at different groups.
