# Supplementary material for: The Cardiotoxicity Induced by Arsenic Trioxide is Alleviated by Salvianolic Acid A via Maintaining Calcium Homeostasis and Inhibiting Endoplasmic Reticulum Stress
Source: Molecules. 2019 Feb 2;24(3):543. doi: 10.3390/molecules24030543 (PMC6384753; doi:10.3390/molecules24030543)
Supplement: Supplementary file 1 [file molecules-24-00543-s001.pdf]

molecules

MDPI

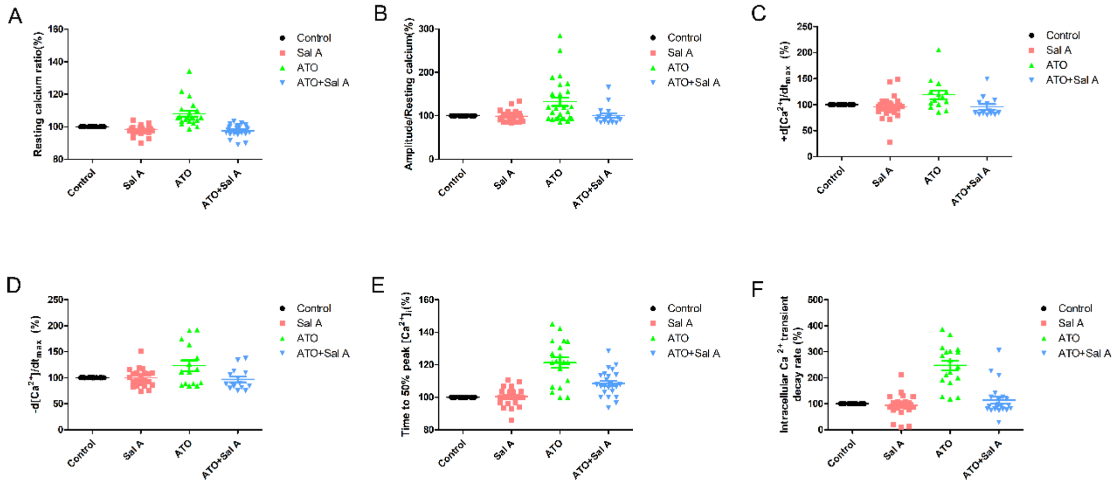

Figure S1. Sal A regulated intracellular Ca^{2+} transients in ARVMs after ATO treatment in dot plots. (**A**) Resting Ca^{2+} ratio. (**B**) Amplitude/resting calcium ratio. (**C**) Maximal Ca^{2+} shortening velocity (+d[Ca^{2+}]/dtmax). (**D**) Maximal Ca^{2+} relaxation velocity (-d [Ca^{2+}]/dtmax). (**E**) Time to 50% peak [Ca^{2+}]$_i$. (**F**) Intracellular Ca^{2+} transient decay rate. n=30-40 per group.

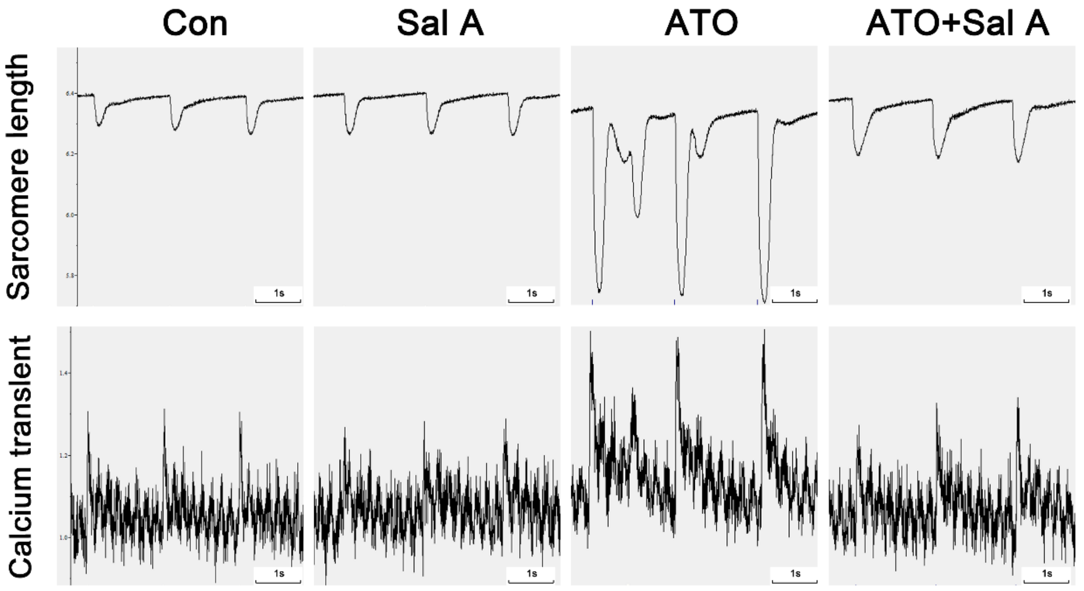

Figure S2. Representative traces of sarcomere shortening and Ca^{2+} transient at different groups.
